# Comparison of Surface Area across the Allograft-Host Junction Site Using Conventional and Navigated Osteotomy Technique

**DOI:** 10.1155/2012/197540

**Published:** 2012-12-18

**Authors:** Ajay Lall, Eric Hohn, Mimi Y. Kim, Richard G. Gorlick, John A. Abraham, David S. Geller

**Affiliations:** ^1^Department of Orthopaedic Surgery, Montefiore Medical Center, The Children's Hospital at Montefiore, Bronx, NY 10467, USA; ^2^The Albert Einstein College of Medicine, Bronx, NY 10461, USA; ^3^Department of Epidemiology and Population Health, Montefiore Medical Center, Bronx, NY 10467, USA; ^4^Department of Pediatrics, Montefiore Medical Center, The Children's Hospital at Montefiore, Bronx, NY 10467, USA; ^5^Rothman Institute, Thomas Jefferson University, Philadelphia, PA 19107, USA

## Abstract

Bulk allograft reconstruction plays an important role in limb-salvage surgery; however, non-union has been reported in up to 27% of cases. The purpose of this study is to quantify average surface contact areas across simulated intraoperative osteotomies using both free-hand and computer-assisted navigation techniques. Pressure-sensitive paper was positioned between two cut ends of a validated composite sawbone and compression was applied using an eight-hole large fragment dynamic compression plate. Thirty-two samples were analyzed for surface area contact to determine osteotomy congruity. Mean contact area using the free-hand osteotomy technique was equal to 0.21 square inches. Compared with a control of 0.69 square inches, average contact area was found to be 30.5% of optimal surface contact. Mean contact area using computer-assisted navigation was equal to 0.33 square inches. Compared with a control of 0.76 square inches, average contact area was found to be 43.7% of optimal surface contact. Limited contact achieved using standard techniques may play a role in the high rate of observed non-union, and an increase in contact area using computer-assisted navigation may improve rates of bone healing. The development of an oncology software package and navigation hardware may serve an important role in decreasing non-union rates in limb salvage surgery.

## 1. Introduction

Allograft reconstruction has become increasingly important as the ability and interest in limb-salvage surgery for the treatment of bone tumors has grown over the past 50 years [1]. Despite its numerous benefits, allograft use has been associated with well-recognized complications, most notably, infection, fracture, and non-union. While infection and fracture may occur either postoperatively or as delayed events, non-union is by definition an early complication, which may be significantly influenced by operative technique.

Although congruous osteotomy cuts are thought to be desirable, exact matching surfaces are rarely achieved using a free-hand technique. This has previously been reported by McGrath et al., who demonstrated that end-cutting intramedullary reamers produced a significantly greater contact area across transverse osteotomies as compared with hand-cutting techniques [2]. With the advent of computer-assisted surgical navigation, whereby increased surgical precision and real-time surgeon feedback is feasible, higher accuracy may be achieved when compared to a freehand technique [3, 4].

Bulk allograft incorporation is likely a complex event, depending upon graft preservation, anatomic location, host vascularity, immunologic host response, and mechanical properties that include the fixation employed and the geometry of the allograft-host junction. [5]. Increased contact surface area across the allograft-host junction has been shown to provide a mechanical advantage, increasing stability as measured by torsional stiffness, maximum torque and maximum displacement [6]. Similarly, contact surface area across the allograft-host junction site may also play an important role in allograft-host junction site healing which, in turn, would serve to lower morbidity and monetary cost associated with non-union.

The purpose of the current study was to quantify average surface contact areas across a simulated allograft-host junction site using both a free-hand and computer-assisted osteotomy techniques. We hypothesized that the computer-assisted technique will result in significantly improved congruity and contact area across the allograft-host junction site.

## 2. Materials and Methods

A 1 cm segment was removed from validated composite femoral sawbones (Pacific Research Laboratories, Vashon, WA) by 2 experienced orthopedic oncologic surgeons, by making two transverse osteotomies using a Stryker System 6 operative sagittal saw (Stryker, Mahwah, NJ) using either a standard free-hand technique or a CT-navigated technique (O-arm Surgical Imaging System, Medtronic, Minneapolis, MN) for real-time intraoperative feedback. Free-hand technique cuts were performed in a conventional manner, with the surgeon using his discretion regarding saw position, angle, and alignment. No jigs or cutting blocks were utilized and other present persons made no input or adjustment. The CT-navigated technique was performed by affixing a 100 mm percutaneous reference navigation pin in the distal metaphysis of the sawbone and attaching the reference frame in routine manner. The O-Arm was used to localize the sawbone by obtaining antero-posterior and lateral fluoroscopic images in order to center and properly position the gantry. A CT scan was then obtained and the imaging was reviewed to ensure adequacy. The saw was navigated by affixing a SUR-TRAC navigation frame and registering the tip of the saw in keeping with the manufacturer's recommendations. Following removal of the osteotomized segment, pressure sensitive paper [Fuji Pressurex Ultralow Film (28–85 PSI, 2–6 kg/cm^2^)] was positioned between the remaining sawbone ends, which served as a simulated allograft-host junction site (SAHJS).

**Figure 1:**
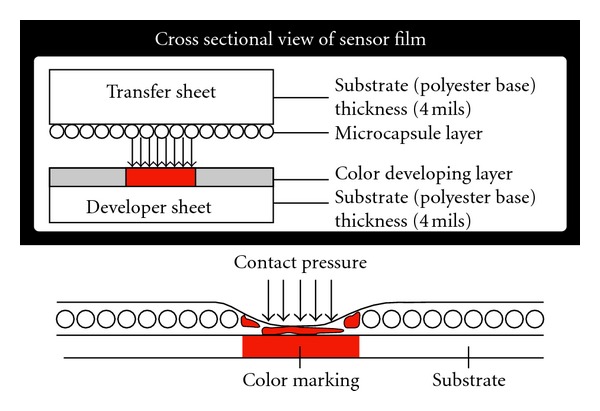
Cross sectional view of PRESSUREX film illustrating mechanism of color mapping from contact pressure.

Pressurex, a mylar based film, contains a layer of microcapsules which upon the application of force are designed to rupture, producing an instantaneous and permanent high resolution “topographical” image of pressure variation across the contact area (Figure 1). Pressure film can be applied between any two surfaces that touch, mate, or impact [7]. The procedure of pressure film application simply involves applying the pressure and removal of the pressure. Similar to Litmus paper, the color intensity of the film is directly related to the amount of pressure applied to it. Pressure ranges being investigated determine the specific type of film used as shown in Table 1.

**Figure 2:**
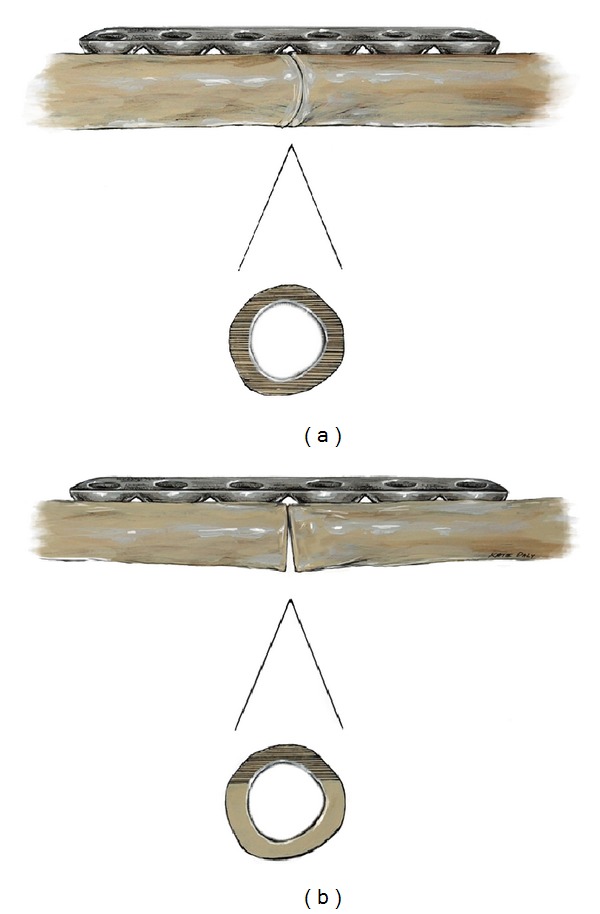
Scheme of femoral sawbones under compression plating at the simulated allograft-host junction site (SAHJS). Images demonstrating 100% contact (a), minimal contact with gapping (b).

Compression across the SAHJS was achieved by first fixing the plate to one side of the SAHJS using a single fully-threaded non-locking cortical screw placed centrally within the plate's hole. Next, an eccentrically placed fully-threaded non-locking cortical screw was inserted on the opposite side of the SAHJS and compression ensued with complete seating of the screw. The pressure indicating film (28–85 PSI, 2–6 kg/cm^2^) acts as a force-sensing resistor between the cut ends of femoral sawbones under compression plating (Figure 2). Hardware was then removed with care in order to protect the pressure sensitive paper from scuffing or manipulation prior to analysis. A total of 32 samples were obtained using the free-hand technique and 22 samples were obtained using computer-assisted navigation. 

Film analysis was performed using the Topaq system, which permits for high resolution full-color representation of pressure distribution along the Pressurex film, serving to represent osteotomy congruity. Utilizing an adapted flatbed scanner, the system scans and interprets the pressure sensitive film to determine the pressure applied at any given point across the surface at resolutions of up to 1000 dots per inch (DPI) [7]. Software statistics and personalized finite element modeling permit for 3D reconstruction of sawbone contact area geometry and provide quantitative values for both contact area and force. 

Control samples were created by applying pressure sensitive film to one end of a perpendicularly cut femoral sawbone using either free-hand or computer assisted navigation. These samples were calculated by assuming that all available surface area across the 2-dimentional cortical surface was in fact utilized and indeed made contact with the opposing bone. This theoretically represents the greatest possible contact area for a transverse osteotomy given the size of the sawbone. Pressurex film was then manually compressed to provide a quantifiable measurement of surface contact. The control sample was defined as the maximum available cortical bone contact surface area and did not include the area representing the intramedullary space. Controls differed, using the free-hand technique (0.69 sq. in.) versus computer-assisted navigation (0.76 sq. in), due to quantitative calculations of the Topaq system. The percent contact area was calculated relative to a control sample with optimal contact area. 

## 3. Statistics

Mean absolute and percentage values were compared between the free-hand and CT-navigated groups with the two sample *t*-test. A two-tailed *P* value of less then 0.05 was considered statistically significant.

## 4. Results

**Figure 3:**
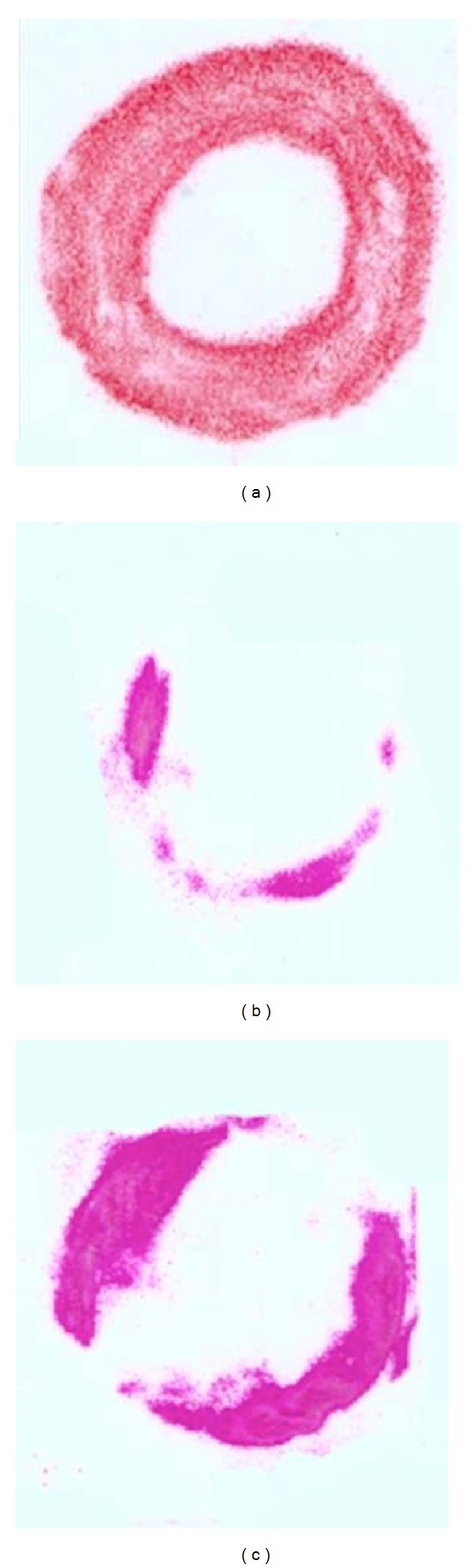
Scanned raw images of PRESSUREX film after contact at the allograft-host junction site made using the free-hand technique. Of the 32 samples, contact area achieved is seen in red for control (ideal) at 0.69 sq. in. (a), minimal contact at 0.07 sq. in. (b), and maximal contact at 0.36 sq. in. (c).

Analysis of the 32 Pressurex samples using the free-hand technique showed a mean contact area of 0.21 sq. in. (range 0.07 to 0.36). As shown in Figure 3, compared with a control of 0.69 sq. in., the mean contact area represents 30.5% of optimal surface contact (range 10.1% to 52.2%) (Table 2). 

**Figure 4:**
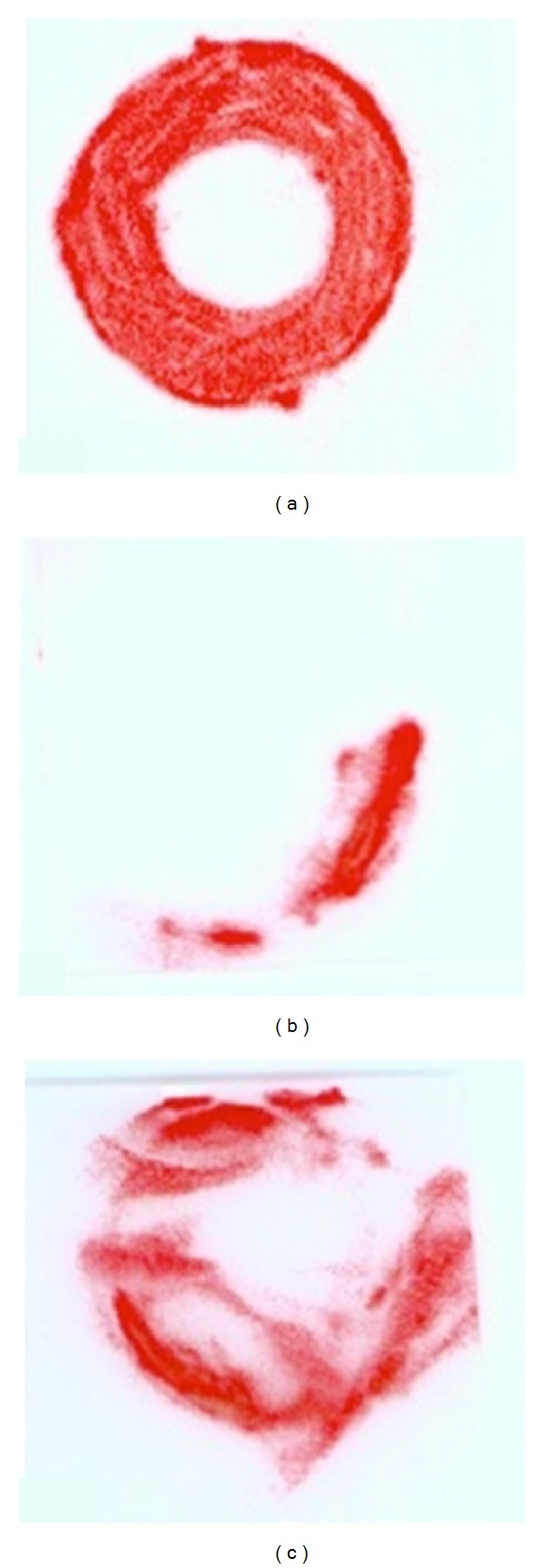
Scanned raw images of PRESSUREX film after contact at the allograft-host junction site made using the computer-assisted navigation technique. Of the 22 samples, contact area is seen in red for control (ideal) at 0.76 sq. in. (a), minimal contact at 0.12 sq. in. (b), and maximal contact at 0.69 sq. in. (c).

Analysis of the 22 Pressurex samples using computer navigation showed a mean contact area of 0.33 sq. in. (range 0.12 to 0.69). As shown in Figure 4, compared with a control of 0.76 sq. in., the mean contact area represents 43.7% of optimal surface contact (range 15.8% to 90.8%) (Table 3).

 A comparison of the two techniques demonstrated the absolute mean contact area was 0.21 sq. in. using free-hand versus 0.33 sq. in. using CT-navigation (*P* = .002). Mean percent contact area was 30.5% using free-hand versus 43.7% using CT-navigation (*P* = .01).

## 5. Discussion

Bone allograft transplantation has played an important role in skeletal reconstruction for more than one hundred and twenty years. As a bone restoring procedure, it provides soft tissue insertions to which host tendon and capsule can be attached and it can delay the need for joint resurfacing for many years. While early challenges revolved around availability of donor bone, recent focus has shifted to safety, bone-banking standards, and the technical processes of donor screening, bone preparation, and storage [8]. Current and future allograft concerns will likely revolve around optimizing union and implant longevity. Non-union, infection, and fracture serve as the major limitations of bulk allograft use.

Nonunion of the allograft-host junction is a well-recognized and well-described complication. Hornicek et al. reported non-union rates ranging from 11%, in patients not receiving chemotherapy, to rates of 27% for patients undergoing chemotherapy [9]. Similarly, in a large series of over 700 allografts spanning 20 years, Mankin et al. reported an overall nonunion rates of 17% [10]. 

Nonunion of allograft-host junctions is a multifactorial event and is both biologically dependent and technique dependent. Allograft bone is not living bone, so osseous union is entirely dependent upon unidirectional healing. This process, creeping substitution, occurs via cutting cones whereby osteoclast-mediated resorption is pursued by osteoblast-mediated bone formation [11–13]. The process is slow and dependent upon intimate contact between allograft-host bone. Gapping, which is tolerated under typical fracture conditions, is likely a substantial barrier to osseous union in allograft-host bone healing [14]. We believe that technical considerations to limit gapping or conversely, maximize bony approximation are thought to be critical. 

Location of non-union plays an important role in that diaphyseal bone has been recognized to heal at a slower rate and have a higher rate of nonunion than metaphyseal bone [15]. Although this discrepancy is likely related to inherent differences between metaphyseal cancellous bone and diaphyseal cortical bone, there is invariably more surface area available for healing within the metaphyseal region, underscoring the relevance of maximizing contact to bone healing.

Modifications in surgical preparation of the bone have been reported. The step-cut is a well-recognized technique, which increases surface area and inherent stability across the allograft-host junction site. However, it is technically more demanding and does not permit for rotational adjustment following the osteotomy, possible reasons which explain why it has become less popular in recent years. Healey et al., 2009, proposed a surgical technique in cases whereby limited remaining bone stock could be supplemented with a structural allograft, which was interposed or telescoped into the remaining host bone [16]. This served to maximize surface contact between host and allograft bone and permit, in turn, use of more conservative prosthesis.

In the current study, computer-assisted navigation was employed, allowing for more accurate transverse osteotomies, thereby increasing contact area at the allograft-host interface. However, even under controlled simulated conditions, absolutely congruent osteotomies are technically difficult to create. Average bony contact areas using navigated techniques were recorded at 43.7%, 13.2% greater than the free-hand technique (*P* = .01). This finding may help explain the observed rate of non-union when using free-hand osteotomy techniques and supports the notion that an increased contact area may promote bone healing.

Limitations of this study include the simulated study design, which may not entirely parallel the intraoperative human condition as well as the small number of samples collected. It is possible that a discrepant amount of bone was lost in one technique compared with the other and that this, in turn, impacted the congruency of the osteotomies. However, since bone loss is largely a function of saw blade cutting characteristics and since the same saw blade design was standardized throughout the study this was felt to be of minimal impact. Our technique of compression plating was standardized as well and intended to recapitulate the intraoperative maneuver whereby a compression screw is fully seated in order to achieve a finite amount of compression across a fracture site or osteotomy site. The compression obtained is not further affected by the placement of additional non-compression screws and for this reason additional screw placement was not deemed necessary. In addition, it is recognized that the deviation from a perfectly congruous osteotomy can take on many orientations and angles. We did not attempt to classify or categorize each sample but rather chose to quantify the resulting contact area as a means of comparing the two techniques. This model does not exactly recapitulate the intraoperative clinical situation in that surrounding soft tissue structures are not present and the entire length of the bone is clearly visualized. This may serve to artificially improve the free hand technique in particular, as adjustments based on visual cues would seemingly be easier. Finally, it is unknown whether the measured improvement in bone-on-bone contact area would in fact translate into improved biologic outcomes of bone-allograft-bone constructs and therefore the clinical relevance remains currently unclear.

In conclusion, although biology and blood supply are most likely more important than perfect mechanical congruency for the successful union at the allograft-host junction site, osteotomies generated with computer-assisted navigation, when compared to free-hand technique, have a significant increase in contact area when apposed. We speculate that increased contact may in turn improve union rates. Likewise, previous studies have reported on enhanced rates of bony union at the allograft-host junction by improvements in contact area [17]. Obvious benefits of this could include decreased morbidity, revision surgery rates, and associated cost. 

Going forward, it is likely that navigated-techniques will play an important role in the planning of, execution of, and reconstruction following complex tumor surgery. Which system and how it is employed or developed remains to be seen. An optimal system would permit for preoperative planning and therefore preoperative custom implant fabrication. In addition, it would allow for easy intraoperative registration, a high degree of accuracy, and easy-to-use user interface. Blending CT and MRI would likely be beneficial as well. Although basic oncology navigation software has recently become available, its continued development and enhancement as well as incorporation of navigated instrumentation such as wide osteotomes and sagittal saw blades are essential.

## Figures and Tables

**Table 1:** Types of pressure sensitive films (Pressurex).

Types	Pressure range
Micro	0.14–1.4 kg/cm^2^
Zero	0.5–2 kg/cm^2^
Ultra low	2–6 kg/cm^2^
Super low	5–25 kg/cm^2^
Low	25–100 kg/cm^2^
Medium	100–500 kg/cm^2^
High	500–1300 kg/cm^2^
Super high	1300–3000 kg/cm^2^

**Table 2:** Freehand osteotomy.

Absolute Value (sq. in.)		Percentage % [(Abs value/Control) 100]	
Mean	0.21	Mean	30.5%
Range	Min: 0.07	Range	Min: 10.1%
	Max: 0.36		Max: 52.2%

Statistical analysis of contact area measured (*N* = 32) using free-hand osteotomy technique. Control = 0.69 sq in.

**Table 3:** Computer-assisted navigation osteotomy.

Absolute Value (sq. in.)		Percentage % [(Abs value/Control) 100]	
Mean	0.33	Mean	43.7%
Range	Min: 0.12	Range	Min: 15.8%
	Max: 0.69		Max: 90.8%

Statistical analysis of contact area measured (*N* = 22) using computer-assisted technique. Control = 0.76 sq in.
